# The role of Anaphase Promoting Complex activation, inhibition and substrates in cancer development and progression

**DOI:** 10.18632/aging.103792

**Published:** 2020-08-15

**Authors:** Cordell VanGenderen, Troy Anthony Alan Harkness, Terra Gayle Arnason

**Affiliations:** 1 Department of Medicine, University of Saskatchewan, Saskatoon, SK S7N 5E5, Canada; 2 Department of Anatomy, Physiology and Pharmacology, University of Saskatchewan, Saskatoon, SK S7N 5E5, Canada; 3 Department of Biochemistry, Microbiology and Immunology, University of Saskatchewan, Saskatoon, SK S7N 5E5, Canada

**Keywords:** Anaphase Promoting Complex (APC), cancer, protein substrates, APC activators, APC inhibitors

## Abstract

The Anaphase Promoting Complex (APC), a multi-subunit ubiquitin ligase, facilitates mitotic and G1 progression, and is now recognized to play a role in maintaining genomic stability. Many APC substrates have been observed overexpressed in multiple cancer types, such as CDC20, the Aurora A and B kinases, and Forkhead box M1 (FOXM1), suggesting APC activity is important for cell health. We performed BioGRID analyses of the APC coactivators CDC20 and CDH1, which revealed that at least 69 proteins serve as APC substrates, with 60 of them identified as playing a role in tumor promotion and 9 involved in tumor suppression. While these substrates and their association with malignancies have been studied in isolation, the possibility exists that generalized APC dysfunction could result in the inappropriate stabilization of multiple APC targets, thereby changing tumor behavior and treatment responsiveness. It is also possible that the APC itself plays a crucial role in tumorigenesis through its regulation of mitotic progression. In this review the connections between APC activity and dysregulation will be discussed with regards to cell cycle dysfunction and chromosome instability in cancer, along with the individual roles that the accumulation of various APC substrates may play in cancer progression.

## INTRODUCTION

The degree to which individuals respond to cancer therapy is highly varied among each cancer patient, reinforcing the belief that each case is heterogeneous and unique. Despite this, research aims to identify common themes and mechanisms of cancer development that could be widely adopted to predict, detect, and target the disease to improve patient outcomes. While an immense variety of cellular malfunctions exist that lead to cancer, there are key, widely accepted, commonalities that serve as hallmarks of cancer [1]. These hallmarks include selective growth and proliferative advantages, altered stress responses, metabolic rewiring, modified vascularization and the ability to invade and metastasize. Cancer cells can also exhibit enhanced genomic instability, a result of multiple mechanisms, including dysregulated DNA synthesis and ineffective mitotic checkpoints [2, 3]. Normally, cells with DNA double strand breaks above a given threshold, generally believed to be determined by p53 [4], would be diverted down the programmed cell death pathway and prevented from replicating [5, 6]. Cancer cells notoriously bypass the usual quality control checkpoints and continue to replicate despite multiple mutations. This persistent damage can then cause a positive feedback loop with promiscuous replication of DNA harbouring damage resulting in further dysregulation of protein function and expression, generating yet greater deregulated cell cycle progression. The ability to continually replicate regardless of excess damage also implies that there is a suppression of apoptotic pathways, which would normally terminate a normal cell undergoing this malignant transformation [7]. While the specific genes altered may differ between malignancies, the defects may produce similar effects, as multiple genes regulate similar pathways.

## The Anaphase Promoting Complex (APC) and cancer development and progression

Oncogenic-like changes (deregulated apoptosis, inadequate quality control of the cell cycle, and accumulated DNA damage) can be influenced by competing stress responsive and nutrient sensing pathways. In the *Saccharomyces cerevisiae* and *Schizosaccharomyces pombe* yeast eukaryotic model systems, a wealth of literature links the antagonistic interactions between the stress and nutrient sensing pathways as critical for genome stability and longevity [8–14]. The Anaphase Promoting Complex (APC) appears to be at a critical nexus point that regulates the molecular equilibrium of these pathways [15–23]. The APC has been observed in yeast to interact with stress response pathways to mediate the response to multiple stresses, with mutations to the APC resulting in genomic instability leading to a variety of phenotypes [15, 16, 18–20, 23, 24]. Indeed, studies using human cell culture show that the APC, when bound by the CDH1 coactivator subunit (APC^CDH1^), controls cell cycle arrest in response to stress [25, 26]. It was observed that APC^CDH1^ inactivation represents the commitment towards cell cycle re-entry. Active APC^CDH1^ facilitates entrance into a quiescent state when stress is encountered, but not when APC^CDH1^ is inactivated. Thus, this provides an explanation for why impaired APC^CDH1^ activity is associated with enhanced genomic instability and cancer progression [27–31], as cell cycle arrest is blocked in the presence of DNA damage, allowing mutations to accumulate.

The APC is a large, structurally and functionally conserved ubiquitin ligase that targets inhibitors of mitotic progression and interphase arrest for ubiquitin- and proteasome-dependent degradation. In humans, the APC is a 1.5 megadalton complex composed of 19 subunits, 15 of which are unique [32]. The yeast complex is equally large with 13 unique subunits, lacking only the human APC7 and APC16. The conserved APC is composed of 3 structural motifs: the platform, the TPR lobe and the catalytic core. The TPR lobe contains many of the subunits targeted for post-translational modifications, while the catalytic core contains APC11, APC2 and APC10 that transfer the ubiquitin molecule from the E2 to the substrate molecule. The platform (APC1, APC4 and APC5) connects the TPR lobe and the catalytic core. The APC, as discussed in this review, interacts with a great number of proteins for proper regulatory control and function. It is also targeted by a variety of signalling networks that phosphorylate, ubiquitinate and acetylate APC subunits, mostly within the TPR lobe, but also APC1. The large structure and intricate assembly is likely required to sort through the many unique, but intertwined signalling mechanisms that control APC activity. APC activity is primarily controlled through exclusive binding by one of 2 activator subunits, CDC20 or CDH1, to form the APC^CD20^ and APC^CDH1^ complexes, respectively; CDC20 promotes anaphase and mitotic progression, while CDH1 regulates mitotic exit and G1 progression [33, 34]. It has been observed that the APC activator and eventual substrate, CDC20, accumulates in many types of cancer cells *in vitro* and *in vivo* [35–38]. This suggests that CDC20-dependent activation of the APC may be a critical component of cancer development and behavior. This is further supported by the observation that expression of both APC2 and APC7 are elevated in acute myeloid leukemia cell lines and patients [39], and that overexpression of APC11 mRNA and protein has been reported in lung cancer cells and patients [40]. Indeed, silencing of CDC20 using RNA interference in pancreatic cells lines augmented cytotoxicity when exposed to chemotherapies [41]. Furthermore, use of the pharmacological agents APCIN or pro-TAME, which inhibit the binding of CDC20 to the APC (and thus APC^CDC20^ formation) resulted in increased apoptosis and death in multiple cancer cell lines, indicating that inhibition of the APC may be a useful anticancer approach [42–44]. Moreover, an interesting recent study showed that cancer cells displaying chromosome cohesion defects were synthetically lethal with APC subunit depletion, providing further evidence that APC inhibition may be a powerful means to killing cancer cells [45]. As well, direct inhibition of the APC by peptides elevated sensitivity of cancer cells to microtubule poisons [46].

Opposing the idea that APC activity is an important driver of cancer development and that inhibiting its activity is a useful approach to treating cancer, are the multiple observations that many APC substrates are elevated in various unrelated cancers, both at their mRNA and protein levels. Many of these substrates are also notable for being markers for poor prognosis [23, 47–51]. The accumulation of these substrates indicates two potential mechanisms; either the accumulation of these proteins leads to APC-independent cancer progression, or it is impaired APC function that leads to the accumulation of multiple substrates and cancer progression. The accumulation of APC targeted mitotic kinases like PLK1, MPS1, and Aurora A/B in cancer has led to efforts to target these molecules for anticancer therapy [52]. However, regardless of *in vitro* successes, lead molecules targeting APC substrates have had limited success in the clinic [53–55]. Nonetheless, while monotherapy may have limited success, these studies reveal that combinatorial treatment with other anticancer drugs shows promise in clinical trials. Thus, the accumulation of multiple APC-targeted proteins in a single cancer cell may be due to a failure of their regulated degradation, suggesting that generalized APC E3 activity may in fact be impaired in cancer cells. Observations that mutations to several APC subunits are associated with cancer progression [56, 57] supports the notion that APC activity may in some cases ward off cancer progression. In addition, the development of small molecule inhibitors of the Spindle Assembly Checkpoint (SAC; inhibits APC activity), TTK/MPS1 protein kinase inhibitor (TTKi) and Mad2 Inhibitor 1 (M2I-1), are observed to be potent anticancer agents *in vitro* [31, 58–62]. In general, the SAC inhibits the APC by sequestering away CDC20 until cells are ready to enter mitosis [63]. SAC inhibitors lead to enhanced APC activity and a shortened mitosis, suggesting that APC activity may be critical for TTKi and M2I-1 anti-cancer function. This was validated by a report showing that cells treated with siRNA against APC subunits APC4 or APC13/SWM1 developed resistance to the SAC inhibitor [31]. This opens the possibility that activation of the APC may enhance cancer treatment by potentially bypassing the spindle assembly checkpoint, pushing highly damaged cells inappropriately into anaphase prior to sufficient DNA repair, causing mitotic catastrophe.

Recent work demonstrates that the aberrant accumulation of many mRNAs involved in the regulation of APC function and mitotic progression in cancer cells are tightly linked, suggesting that the APC plays a general role in protecting against cancer development and/or progression. It was observed that the accumulation of CDC20 in tissues from a variety of unrelated malignancies was associated with a cluster of 139 genes that were likewise also markedly overexpressed. Many of the genes in the CDC20-associated gene signature defined genes involved in cell proliferation, DNA damage response, and chromosome segregation [37]. This CDC20-associated gene set was originally found overexpressed in glioma transcriptomes, and was found to be a robust predictor of poor clinical prognosis in over 1,000 patient datasets investigated. This adds further support for the notion that APC function may be a critical trigger for the development and progression of multiple cancers.

## APC function

**Figure 1:**
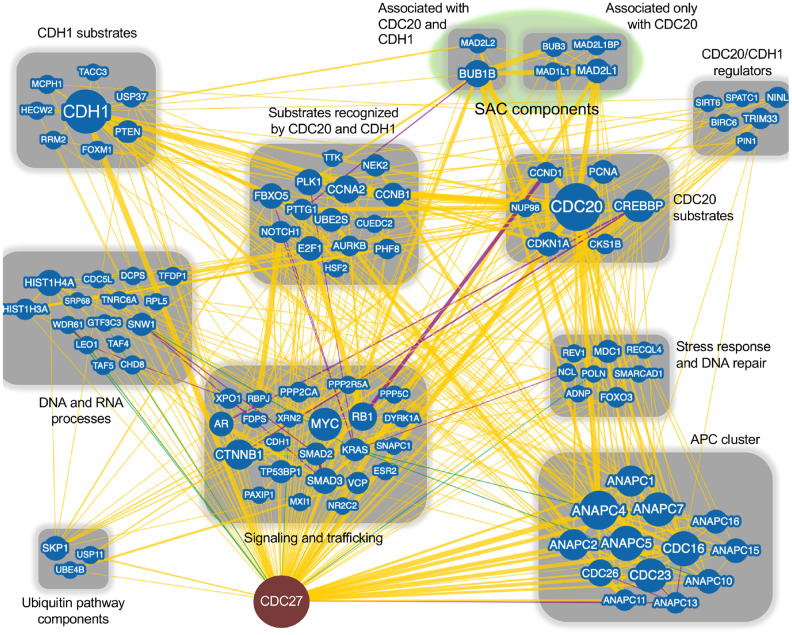
**CDC27 network interactors**. The BioGRID database was searched for CDC27 interactors. The search resulted in 144 protein nodes (blue circles) that interact with CDC27, producing 602 physical edges (yellow lines), 16 genetic edges (green lines) and 18 physical/genetic edges (purple lines). An edge is the line connecting 2 proteins. Many proteins have multiple interactors, generating multiple edges for a single protein. The search was done with the minimum evidence filter set at 1 (see Supplementary Figure 1 for raw data). Proteins that only interacted with CDC27 (1 edge) were lost when the filter was set at 2 and were not included in this analysis. Each node was manually manipulated for this clustering exercise.

The APC is most often considered in terms of its mitotic functions. However, there are many ancillary functions that are performed by the APC including: maintaining genomic stability [19, 64–66], regulating interphase progression [67–69] and apoptosis [70, 71]. Dysregulation of these additional functions can be found in cancer. Both of the APC coactivators have tumor related functions; CDC20 is a well-known oncogene which drives improper cell proliferation [36, 49, 72–74], while CDH1 is considered a tumor suppressor that regulates mitotic exit, entrance to S phase, induces quiescence under stress conditions and maintains genomic stability [16, 66, 75, 76]. We performed a BioGRID analysis of CDC27 to begin to understand the network differences between CDC20 and CDH1, as CDC27 is the key entry point for the coactivators; CDC27 recruits both CDC20 and CDH1 into the APC [77, 78] (Figure 1). BioGRID is a biological database detailing protein-protein, genetic and chemical interactions, as well as post-translational modifications (https://thebiogrid.org). This analysis revealed 144 unique nodes for CDC27, with 602 physical edges, 16 genetic edges and 18 combined physical/genetic edges. Each node, which defines a different gene, was searched on PubMed for interactions with the APC, with APC substrates identified that were uniquely targeted for degradation by CDC20 and/or CDH1. CDC27 was also found to interact with clusters of signalling and trafficking molecules, stress response and DNA repair proteins, CDH1/CDC20 regulators, SAC components, and proteins involved in DNA and RNA processes. This variety of interactors validates the many roles the APC has been described to fulfill.

**Figure 2:**
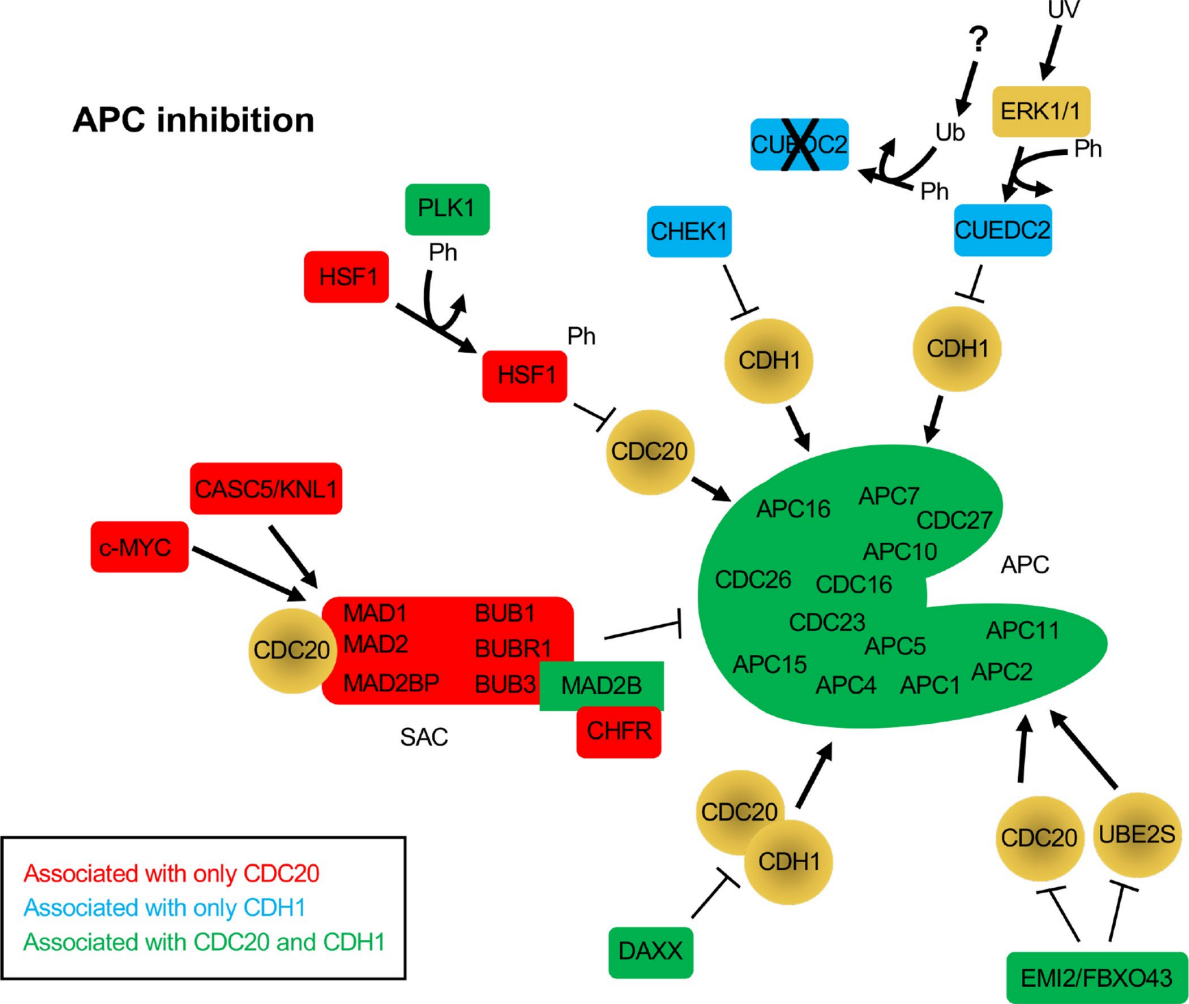
**Protein inhibitors of the APC that function through CDC20 and/or CDH1.** The BioGRID database was separately searched for CDC20 and CDH1 interactors. To avoid confusion with the cadherin 1 gene (also called CDH1), the alias FZR1 was used to search for the CDH1 coactivator. 181 nodes were identified for FZR1, identifying 801 physical, and 18 genetic edges (see Supplementary Figure 2). For CDC20, 175 nodes were identified, with 911 physical edges and 8 genetic edges (see Supplementary Figure 3). All protein nodes identified were searched using PubMed. Proteins found to inhibit the APC, but not serve as substrates, are shown here. Proteins unique to CDC20 are shown in red, those unique to CDH1 are shown in blue, and those identified in both searches are shown in green. All APC subunits were identified in both searches. Ph, phosphorylation; Ub, ubiquitination.

**Figure 3:**
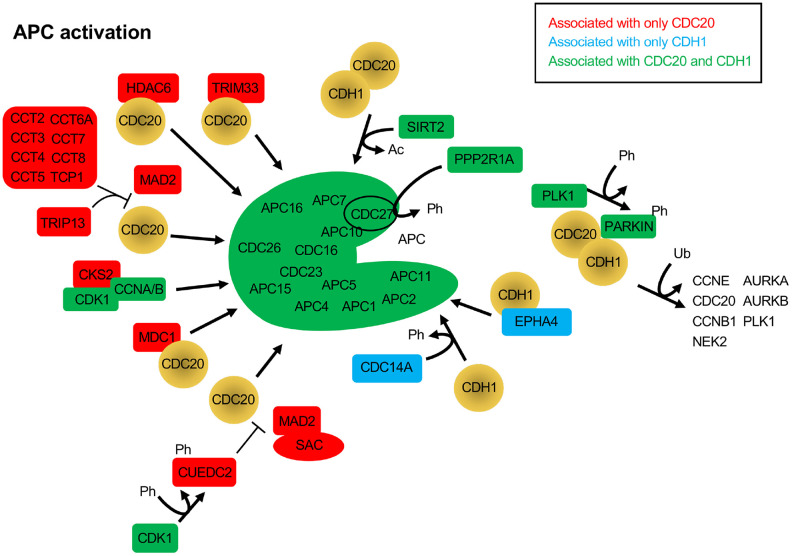
**Protein activators of the APC that function through CDC20 and/or CDH1.** The BioGRID database was searched for CDC20 and CDH1 interactors. All protein nodes identified were searched using PubMed. Proteins found to activate the APC, but not serve as substrates, are shown here. Proteins unique to CDC20 are shown in red, those unique to CDH1 are shown in blue, and those identified in both searches are shown in green. PARKIN, when phosphorylated by PLK1, is believed to recruit CDC20 and CDH1 to ubiquitinate APC substrates. Ph, phosphorylation; Ac, acetylation; Ub, ubiquitination.

**Figure 4:**
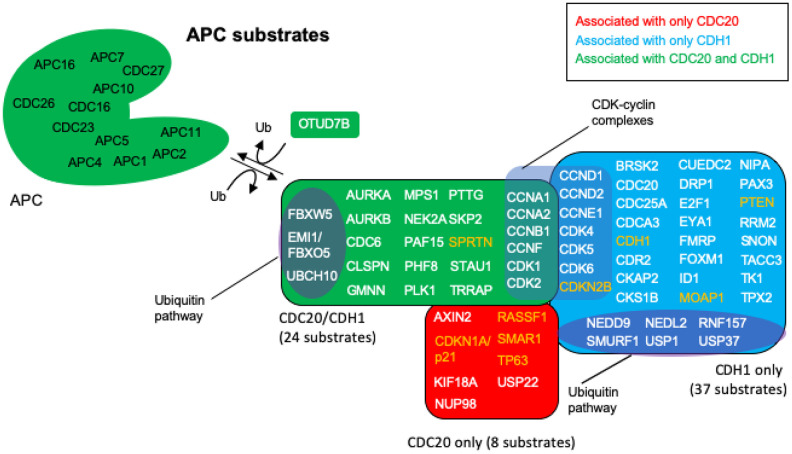
**APC substrates that are unique to CDC20 or CDH1, and those that are acted on by both.** The BioGRID database was searched for CDC20 and CDH1 interactors. All protein nodes identified were searched using PubMed. Proteins found to serve as APC substrates are shown here. Proteins unique to CDC20 are shown in red, those unique to CDH1 are shown in blue, and those identified in both searches are shown in green. Subgroups within the clusters are highlighted for those involved in the ubiquitin pathway, and those composing CDK-cyclin complexes. Proteins highlighted in white are proteins overexpressed in cancers with oncogenic potential, while those highlighted in orange are mostly downregulated in cancers showing potential tumor suppressing activity. 8 proteins are uniquely targeted for degradation by CDC20 and 37 by CDH1, while 24 protein substrates are shared by both, for a total of 69 potential substrates. The deubiquitinase OTUD7B that deubiquitinates APC substrates was identified in both searches.

Subsequent BioGRID searches were performed for CDC20 and CDH1 separately to specifically identify common and unique interactors for the 2 coactivators. One hundred and eighty one and 175 interaction nodes were identified for CDH1 and CDC20, respectively, resulting in 819 edges for CDH1, and 919 edges for CDC20. Nodes define proteins interacting with CDC20 or CDH1, while an edge is a line that connects any 2 proteins. A protein node may have more that 1 edge, resulting in more edges than nodes. Thirteen APC subunits were identified by both the CDC20 and the CDH1 searches. Each protein node was searched on PubMed to identify overlaps with APC function. Any protein that did not overlap with the APC on PubMed was not followed further. Physical interactions identified by BioGRID can be part of global screens where individual hits are not discussed in manuscripts, so are not picked up in PubMed searches. So, while these proteins likely physically associate with CDC20 and/or CDH1, not enough information is available to discern the mechanism of association. Further, many proteins may not be direct interactors, but interact through intermediaries defined by complexes. For this analysis we focused on proteins involved in APC inhibition (Figure 2), APC activation (Figure 3) or are potential APC substrates (Figure 4).

## APC inhibition

A number of proteins were identified that interacted with either or both CDC20 and CDH1 that were not observed as substrates, but were involved in APC inhibition. The SAC components MAD1, MAD2, MAD2BP, BUB1, BUBR1 and BUB3 were all identified only in the CDC20 search, while MAD2B was identified in both searches. As discussed above, the SAC blocks CDC20 from interacting with and activating the APC [63]. Three different proteins were specifically identified in the CDC20 search that work with the SAC to suppress APC activity, c-MYC, CASC5/KNL1 and CHFR (Figure 2). c-MYC was shown to drive the expression of MAD2 and BUBR1, which corresponded to chromosome instability and DNA strand breaks as a result of impaired repair of replication-stress induced DNA lesions in G2 [79]. In addition, the protein CDR1, an APC^CDH1^ substrate, binds c-MYC to activate its transactivation; elevated accumulation of CDR1 in cancer cells as a result of APC inhibition or defect promotes c-MYC oncogenic function [80]. The protein encoded by CASC5/KLN associates with the SAC to provide a scaffold for protein complex assembly. KNL is phosphorylated by MPS1, a SAC checkpoint kinase that is also an APC substrate, which enables KNL to recruit BUB1-BUB3-BUBR1 to unattached kinetochores and inhibit APC activity [81]. The CHFR protein, described as a tumor suppressor, also promotes the SAC and APC inhibition by facilitating the MAD2-CDC20 interaction [82].

Several other proteins were identified in the CDC20 and CDH1 searches that function as APC inhibitors (CHEK1, CUEDC2, HSF1, DAXX, EMI1/FBXO5 and EMI2/FBXO43). CHEK1 depletion results in disruption of CDC20 and MAD2 localization to kinetochores and decreased CDC20 and MAD2 protein levels [83]. This suggests that CHEK1 is required for APC inhibition. A second study describes this further as it shows that CHEK1 inactivates APC^CDH1^ in the presence of replication stress by targeting CDH1 for degradation, thereby inhibiting APC activity [84]. CUEDC2 is an interesting protein that functions to inhibit APC^CDH1^, yet activate APC^CDC20^. In G1, CUEDC2 binds to and inhibits APC^CDH1^, thereby stabilizing Cyclin A and promoting G1-S transition [85]. This is blocked by UV irradiation. In the presence of UV, ERK1/2 phosphorylates CUEDC2, leading to ubiquitin and proteasome dependent degradation. The E3 responsible for CUEDC2 degradation has not yet been identified. Activation of APC^CDC20^ by CUEDC2 is discussed below. HSF1 functions in cancer by inhibiting the interaction of CDC20 with CDC27 and blocking APC activation [86]. The overproduction of HSF1 resulted in the accumulation of APC substrates, inhibited mitotic exit and generated aneuploidy. It was also found that HSF1 phosphorylation by PLK1 was required to bind CDC20 and inhibit APC activity [86]. There are 2 additional APC inhibitors called Early Mitotic Inhibitors (EMI) 1 and 2. EMI1 acts as both an inhibitor and an APC^CDH1^substrate [25, 26]. EMI1 levels are kept low during G1 by APC^CDH1^, and then high during S and G2 when APC activity is low. APC inactivation is triggered by CDK2/Cyclin E activity during G1, which coincides with increased *EMI1* mRNA expression, which serves to maintain APC inhibition. EMI2, on the other hand, works by inhibiting the interaction of the APC with its E2 component UBE2S in unfertilized Xenopus eggs, thereby blocking unfertilized eggs in metaphase of meiosis II [87]. Upon fertilization, EMI2 is targeted for degradation by the SCF^β-TrCP^ complex. EMI2 also blocks APC activity by blocking the association of CDC20 with the APC [88]. Lastly, the DAXX protein is often observed to be overexpressed in prostate cancer cells. DAXX encodes APC recognition motifs called destruction boxes. DAXX interacts with both CDC20 and CDH1 via these motifs but does not appear to be a substrate [89]. This interaction is sufficient to disrupt APC function.

## APC activation

The CDC20 and CDH1 BioGRID searches also revealed proteins that have not yet been identified as substrates, but have APC activation potential. When SAC activity is no longer required, the complex of MAD2, BUBR1 and BUB3 bound to CDC20 must be disassembled. This process requires ATP, and a number of ATP-dependent activities have been described to assist in the dissolution of the SAC, such as TRIP13, p31^comet^ and the CCT chaperonin [90]. p31^comet^ was not identified in the BioGRID searches, but peptides derived from p31^comet^ have been developed in yeast that bind to the APC and disrupt interaction of CDC20 and CDH1 with the APC [46]. Both TRIP13, and 8 components of the CCT chaperonin (CCT2, CCT3, CCT4, CCT5, CCT6A, CCT7, CCT8 and TCP1) were specifically identified only in the CDC20 search (Figure 3). The CCT chaperonin binds CDC20 and is a necessary factor promoting CDC20 binding to the APC [91]. It was observed that the combined action of the CCT chaperonin with TRIP13 is sufficient to completely disassemble the SAC [90]. TRIP13 has been found to interact with p31^comet^ to induce checkpoint silencing and localizes to kinetochores [92]. Overexpression of TRIP13 is observed in cancers with poor prognosis and is associated with chromosome instability believed to be due to premature checkpoint silencing.

A number of additional APC activators were specifically identified in the CDC20 search: CKS2, CUEDC2, HDAC6, MDC1 and TRIM33 (Figure 3). CKS2 is a Cell division cycle Kinase Subunit that binds to the CDK1/Cyclin A/B complexes to promote their cell cycle driving function. CKS2 is required for meiosis in mammalian cells and mice lacking CKS2 show reduced meiotic development and defective APC^CDC20^ function [93]. As written above, CUEDC2 inhibits APC^CDH1^, but can also activate APC^CDC20^. During mitosis CUEDC2 is phosphorylated by CDK1 [94]. This allowed phosphorylated CUEDC2 to bind to CDC20, and facilitate its release from the SAC component MAD2, thus activating APC^CDC20^. In another study, APC^CDC20^ was found to be important for neural development by playing a role in post-mitotic dendrite morphogenesis [95]. This unique APC^CDC20^ activity was facilitated by HDAC6, a histone deacetylase that is localized to centrosomes, along with CDC20 in neurons. HDAC6 was required for the polyubiquitination of CDC20, and the activation of APC^CDC20^, driving the differentiation of dendrites. MDC1 is a mediator of a DNA damage checkpoint, and was shown to interact specifically with CDC27 [96]. This interaction required phosphorylated CDC27 and was driven by DNA damage. A subsequent study showed that loss of MDC1 resulted in a mitotic arrest that was BUBR1 and ATM signalling independent [97]. Cells lacking MDC1 had impaired APC activity, reduced CDC20 levels, and failure of remnant CDC20 to bind the APC. TRIM33 is a member of the RING (really interesting new gene) domain E3 ligases, and has been described as a transcriptional corepressor involved in SMAD4 signaling [98, 99]. TRIM33 has also been shown to interact specifically with APC^CDC20^ and is a component of the mitotic checkpoint complex (MCC), a complex of MAD1, MAD2, BUBR1, BUB3 and CDC20 [100]. The interaction of TRIM33 is complex, as it was shown that TRIM33 will still bind APC in the absence of CDC20, but will not bind APC^CDH1^. Further, binding assays revealed that TRIM33 only associated with MCC-APC when the SAC was active, not once it was satisfied. This was interpreted to suggest that TRIM33 is required to promote APC^CDC20^ function once the SAC is inactive.

The phosphatase CDC14A and the receptor tyrosine kinase superfamily member EPHA4 both activate the APC through interactions with CDH1. CDH1 is phosphorylated by CDK/Cyclin B complexes, which blocks interaction of CDH1 with the APC. Dephosphorylation of CDH1 by Cdc14 in yeast and CDC14A in mammalian cells relieves the inhibitory pressure and enables APC^CDH1^ activation [101]. However, CDC14A does not influence APC^CDC20^ function. The EPHA4 receptor is involved in neural homeostatic plasticity through interactions with APC^CDH1^ [102]. Elevated synaptic activity triggers the tyrosine phosphorylation of EPHA4, which then interacts with APC^CDH1^ to target GLUR1 for degradation to reduce synaptic signalling.

Two additional proteins promote mitotic progression by interacting with both CDC20 and CDH1, but in different ways, SIRT2 and PARKIN. SIRT2 is a protein deacetylase and is a member of the Sir2 family of deacetylases. Sir2 was first studied in yeast as a histone deacetylase, and was shown to have a conserved role in promoting longevity in model systems [103, 104]. SIRT2 has been shown to provide anti-tumor potential by deacetylating both CDC20 and CDH1 to promote their recruitment to the APC and cell cycle progression [65]. Loss of SIRT2 in mouse embryonic fibroblasts (MEFs) resulted in stabilized APC substrates, centrosome amplification, and aneuploidy, with mice lacking SIRT2 experiencing increased tumor development. PARKIN, on the other hand, is a RING domain E3 family member that is capable of mono- and polyubiquitinating substrates, with neuroprotective and tumor suppressor potential [105]. Interactions with the APC coactivators were revealed in a study where MEFs lacking PARKIN were shown to have mitotic defects and high levels of multiple APC substrates, such as PLK1, Aurora A, Aurora B and Cyclin B1, for example [106]. This work also revealed that PARKIN forms complexes with either CDC20 or CDH1 that were independent of the APC. Interestingly, depletion of both PARKIN and APC11 recapitulated CDC20 depletion, whereas depletion of PARKIN or APC11 only partially impaired Cyclin B1 degradation. Taken together, it is apparent that there are multiple complex mechanisms in play to regulate APC function. Shifts in the equilibrium of this balancing act could have significant impacts on cell health and viability.

## APC substrates

The nodes identified in the BioGRID analyses of CDC20 and CDH1 were all searched by PubMed for any relationship to “anaphase promoting complex”. The resulting literature was assessed for any signs that the particular protein was unstable and targeted for degradation by either APC^CDC20^ or APC^CDH1^ or both. This search revealed that 69 of the identified proteins were associated with literature related to degradation by the APC (Figure 4; Table 1). Reviews have been written recently that list APC substrates (25 [107], 46 [32], 16 [108], 13 [38]), but the 69 potential substrates identified here, to the best of our knowledge, is the largest cohort of APC substrates assembled. Literature for the proteins identified here as substrates that were not in previous lists are cited in Table 1 [32, 38, 67, 80, 85, 107–142]. Eight proteins were identified only in the CDC20 BioGRID search, 37 identified only in the CDH1 search, and 24 as targeted by both. OTUD7B was identified in both searches and acts as a cell-cycle regulated deubiquitinase that counters APC function [143]. Confirmation for 5 of the proteins, CCND2, CDK1, CDK2, CDK6 and CDKN2B could not be obtained in the literature. The APC targets CCND1, CDK4, CDK5 and CDKN1A/p21 for degradation, CDK1/2/6 all associate with cyclins that are targeted by the APC, and CKDN2B is a CDK4/6 inhibitor that physically interacts with CDK4/6 [38, 118, 120–122, 134–136]. These proteins are likely substrates, but confirmation requires further analyses. As discussed above, APC^CDC20^ and APC^CDH1^ are believed to play opposed roles in cancer development, with APC^CDC20^ thought to play an oncogenic role [35–38], and APC^CDH1^ playing a tumor suppressive role [47–51, 56–62]. To gain further insight into these observations we searched each protein in the APC substrate list for a role in cancer using PubMed. All 69 of the putative substrates have been described as being involved in cancer progression. Of the 69 proteins identified in cancer searches, 9 were described as tumor suppressors (orange lettering in Figure 4) and 60 as possible tumor promoters (white lettering in Figure 4). This suggests that proper APC activity is responsible for the targeted degradation of 60 proteins found elevated in tumors and 9 found reduced in tumors. If CDC20 is involved in tumor formation, then we expected that the bulk of the tumor suppressors targeted by the APC would rely on CDC20 activity, whereas the tumor promoters should be specifically targeted by CDH1. As shown in Figure 4, 4 of the 8 proteins potentially targeted by only APC^CDC20^ are described as tumor suppressors in the literature, while 33 of the 37 proteins potentially targeted only by APC^CDH1^ are described as oncogenes. Of the 24 proteins potentially targeted by both, all but one has been described as elevated in tumor cells. These observations add significant weight to the idea that the APC plays a critical role in cancer development. It is also clear that the APC could potentially be involved in both tumor promotion and tumor suppression, depending on the activity equilibrium between APC^CDC20^ and APC^CDH1^.

**Table 1:** Human APC substrates identified from BioGRID CDC20/CDH1 queries and PubMed searches of resultant hits.

**CDC20 specific**	**CDH1 specific**	**shared by CDC20 and CDH1**
AXIN2 [38]	BRSK2 [117]	AURKA [32, 107]
CDKN1A/p21 [38]	CCND1 [120]	AURKB [32, 108]
KIF18A [109]	CCND2 (?)	CCNA1 [32, 107]
NUP98 [110]	CCNE1 [119]	CCNA2 [32]
RASSF1 [107, 113]	CDC20 [32, 107]	CCNB1 [32, 107]
SMAR1 [114]	CDC25A[32, 107]	CCNF1 [67]
TP63 [115]	CDCA3 [32]	CDC6 [32, 107]
USP22 [116]	CDH1/FZR1 [32]	CDK1 (?) - interacts with Cyclin B1 [134]
	CDK4 [118]	CDK2 (?) - interacts with Cyclin E1 [135]
	CDK5 [121]	CLSPN [136]
	CDK6 (?) - interacts with Cyclin D1 [32]	EMI1/FBXO5 [25]
	CDKN2B (?) - interacts with CDK4/6 [122]	FBXW5 [137]
	CDR2 [80]	GMNN [32, 107]
	CKAP2 [107]	MPS1/TTK [138]
	CKS1B [32]	NEK2 [107]
	CUEDC2 (?) [85]	PAF15 [111]
	DRP1 [123]	PHF8 [139]
	E2F1 [107]	PLK1 [32, 107]
	EYA1 [32]	PTTG [32, 107]
	FMRP [108]	SKP2 [32]
	FOXM1 [32, 107]	SPRTN/DVC1 [140]
	ID1 [32, 108]	STAU1 [141]
	MOAP1 [124]	TRRAP [142]
	NEDD9 [125]	
	NEDL2 [126]	
	NIPA [127]	
	PAX3 [128]	
	PTEN [129]	
	RNF157 [130]	
	RRM2 [32]	
	SMURF1 [131]	
	SNON [132]	
	TACC3 [133]	
	TK1 [32, 107]	
	TPX2 [32]	
	USP1 [107, 112]	
	USP37 [32]	

## Normal activation and activity of the APC E3 Ligase during mitosis

The APC targets specific proteins for ubiquitin- and proteasome-dependent degradation, with as many 69 different proteins serving as targets (see Figure 4). These proteins are found in different tissues at different times, involved in a variety of mechanisms required for mitotic progression and overall cell health, and are defined by specific encoded motifs. The primary motif of proteins targeted by the APC is the destruction box (D-box, RxxLxxI/VxN), which exists on a multitude of APC substrates and is targeted by both APC^CDC20^ and APC^CDH1^ [144–146]. Both coactivators contain a WD40 domain that binds APC substrates [146], and assists with APC and E2-ubiquitin interactions to promote APC E3 activity [147–149]. A variety of secondary motifs are recognized by either APC^CDH1^ or APC^CDC20^ including the KEN box (KENxxD) [145] and L box (LXEXXXN) [19], which are targeted by APC^CDH1^, and an LR motif which is targeted by APC^CDC20^ [109]. These secondary motifs act to target specific proteins [42]. Subunits critical for APC E3 ubiquitin ligase function include APC2 and APC11 which perform the catalytic activity (APC11 encodes the RING domain subunit containing the catalytic cysteine for ubiquitination) [140]. The APC3/CDC27 and APC8/CDC23 subunits bind to the CDC20 and CDH1 coactivator proteins [150, 151], while the APC10 subunit is involved in substrate recruitment within the inner cavity of the APC structure in collaboration with the coactivator subunits [152].

During metaphase, the spindle assembly checkpoint (SAC, composed of MAD1, MAD2, BUBR1, and BUB3) is active, delaying mitotic progression until all sister chromatids are securely attached to the mitotic spindle [153]. The SAC is maintained by the MCC, a multi-subunit complex that inhibits APC activity until all kinetochores are properly secured to a microtubule [109]. The MCC component MAD2, when associated with the kinetochore via MAD1, binds to the N-terminus of CDC20, which then associates with BUBR1 and BUB3 to form the tetrameric MCC. The MAD2-inhibitor, M2I-1, functionally disrupts the MAD2-CDC20 interaction, freeing CDC20 for subsequent APC activation [58]. Recent cryo-EM studies revealed that the MCC complex binds two CDC20 molecules, suggesting that MCC also interacts with CDC20 bound to APC. In the cryo-EM structure, MCC-CDC20 binds to APC^Cdc20^, where MCC-CDC20 occupies the large APC^Cdc20^ central cavity [154–156]. BUBR1 interacts with both CDC20 molecules, thereby disrupting the ability of both CDC20 molecules to bind substrate. This occurs because BUBR1 encodes D-box and KEN-box APC recognition motifs, through which CDC20 binds [157]. Once microtubules are properly attached to the kinetochores associated with chromosomes, the SAC becomes inactivated and CDC20 is released from the SAC so it can in turn activate the APC [158]. There are multiple molecular networks that work together to ensure that the SAC is properly regulated in both positive and negative manners (see Figures 2, 3).

Once the SAC is inactivated, the first of two phases of APC activity relevant to mitosis begins, where the APC promotes anaphase by the ubiquitination (and subsequent proteasomal degradation) of multiple protein targets. Two prominent proteins involved in chromosomal segregation are Securin (encoded by *PTTG1*, which is targeted by the APC for degradation) and Separase (which is not directly targeted by the APC). Securin is an inhibitory chaperone of Separase, which acts by allosterically altering the conformation of bound Separase to prevent binding to target proteins [159]. Separase is a cysteine protease that cleaves the kleisin subunit of cohesin. Cohesin acts to bind sister chromatids together and cleavage of the kleisin subunit results in dissolution of the cohesin ring binding sister chromosomes together, inducing chromosomal segregation [160, 161]. The APC acts by polyubiquitinating Securin, targeting it for degradation, and enabling Separase activity. The newly activated Separase then triggers chromosomal segregation by cleaving the cohesion kleisin subunit.

While bound to CDC20 the APC will also self-regulate in a negative feedback loop where it targets Cyclin B1 for degradation. At the G2/M transition Cyclin B1 is synthesized to initiate anaphase. Cyclin B1 functions by binding and activating cyclin dependent kinase 1 (CDK1), which phosphorylates multiple targets to drive anaphase, including APC subunits and CDH1 [162, 163]. The Cyclin B1/CDK1 complex is crucial for phosphorylating APC subunits in a manner that promotes APC^CDC20^ activity while inhibiting interaction of CDH1 with the APC. Thus, the degradation of Cyclin B1 results in the loss of phosphorylation of many targets, including APC subunits, allowing for the replacement of CDC20 by CDH1 [162, 163]. The incorporation of CDH1 into the APC initiates the targeting of a new suite of protein degradation targets and the second phase of APC activity that permits a regulated mitotic exit and maintenance of G1 progression. These targets include, amongst others, CDC20 and FOXM1, and residual Cyclin B1 (Figure 4; Table 1), which a great deal has already been written (for example, see [16, 107, 164]). The role of the APC in regulated mitotic progression and G1 maintenance is essential for the maintenance of chromosomal integrity and genomic stability [76, 165]. Loss of chromosomal integrity drives the heterogeneity of malignant cells and may help promote changes in cancer biology resulting in the acquisition of multiple-drug resistance, metastatic, or other characteristics [166–170].

## Dysregulation of CDC20 or CDH1 impacts APC activity and cancer biology

**Figure 5:**
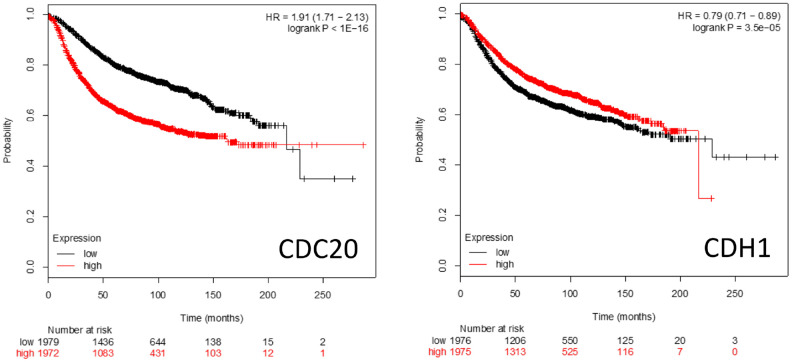
**Kaplan-Meier survival plots comparing high vs low expression of the APC coactivators CDC20 and CDH1 mRNAs in breast cancer patients.**

Kaplan-Meier survival plots (https://kmplot.com/analysis/) of patient survival rates when either CDC20 or CDH1 are over- or underexpressed is shown in Figure 5. High level CDC20 expression is associated with poor patient survival rates, whereas high level CDH1 expression is associated with a slightly better survival rate. This is consistent with the literature suggesting that CDC20 and CDH1 interact with a distinct cohort of proteins and pathways (Figures 1–4) and have distinct roles in cell homeostasis when associated with the APC.

### CDC20

CDC20 has been identified as being an oncogene [38]. Both overexpression and augmented protein abundance have been correlated with poor prognosis for several unrelated cancer types including brain astrocytoma [72], gastric [171], breast [49], colorectal [172], prostate [36], and pancreatic cancers [74]. A recent study found that patients with overexpression of BUB1B, CDC20, CCNA2 and CDK1 were more likely to exhibit the worst cancers [173]. Increases of CDC20 drive a dysregulated mitotic cycle in part by overwhelming the inhibitory capacity of the SAC; while the SAC is capable of blocking CDC20 function while bound to the APC, it can only simultaneously sequester 2 molecules of CDC20 at a time [154, 158]. Overaccumulation of CDC20 could forcibly activate the APC, despite an active SAC, to drive the cell through an unregulated mitotic cycle (referred to as mitotic slippage) and result in dysregulated proliferation [174, 175]. One obvious mechanism leading to CDC20 accumulation would be the dysfunction of the APC itself, resulting in inefficient CDC20 degradation. However, inhibitory mutations within Speckly-type POZ Protein (SPOP) may also result in CDC20 accumulation, as SPOP promotes the E3 ligase activity of Cullin proteins that contribute to CDC20 polyubiquitination and subsequent degradation [73]. It is thought that by driving improper APC activity (and therefore mitosis) that CDC20 primarily contributes to tumorigenesis. However, the observation that overexpression of *CDC20* is accompanied by the overexpression of a host of other genes associated with APC impairment in other cancers [37], including overexpression of other APC substrates (Figure 4), indicates that it may be APC impairment, not specifically CDC20 overexpression, that is important for cancer development and/or progression, in at least some cases.

### CDH1

The potential role that CDH1 plays in cell biology and tumor development is different from CDC20, as it appears to act as a tumor suppressor [42]. Loss of CDH1 activity is a common occurrence in cancer development, and the generation of heterozygous CDH1^+/-^ mice that are haploinsufficient incur greater rates of cancer formation [176–178]. This indicates an overall tumor suppressive function. Loss of CDH1 activity generates chromosomal abnormalities [75, 76, 176, 179], elevated sensitivity to DNA damage [180, 181], insufficient loading of Mini-Chromosome Maintenance proteins (MCMs) [75], and premature S phase entry [67, 75, 182, 183]. These abnormalities are a result of loss of key CDH1 functions when underexpressed, which include cell cycle arrest upon nutrient and genotoxic stress [16, 177, 184–186], regulation of S phase entrance [67, 164, 183], and promoting mitotic exit [187, 188]. CDH1 delays S phase progression until the cell is prepared for DNA replication by targeting proteins involved in DNA replication and S phase progression for degradation, such as Cyclin F, SKP2 (subunit of the SCF ubiquitin ligase), ORC1, CDC6 and RRM2 [32, 67]. Three activities have been reported to decrease CDH1 protein levels as cells approach S phase: APC^Cdh1^ autoubiquitination [189, 190], SCF^CycF^ [67] and SCF^βTRCP^ [191]. The complicated relationship between *CDH1* and cancer progression was described when suppression of *CDH1* in B cell acute leukemia initially resulted in mitotic catastrophe and apoptosis, but long-term *CDH1* loss contributed to development of treatment resistance [192]. It was also reported that *CDH1* was found overexpressed in many malignant tumor samples, along with other APC substrates [47].

CDH1 accretion may also promote cancer development and progression. CDH1 works antagonistically with the SAC and can act to induce mitotic slippage. [193, 194]. APC^CDH1^ overactivity from either CDH1 overexpression, or loss of the APC^CDH1^ inhibitor, early mitotic inhibitor 1 (EMI1), may also result in DNA re-replication through the over-degradation of Geminin [195, 196]. In G2 and S phase, Geminin acts to inhibit CDT1, which is responsible for initiating DNA replication. Therefore, inappropriate loss of CDT1 inhibition may result in DNA replication occurring multiple times, triggering aneuploidy [195–197]. The wide variety of CDH1-associated activities demonstrates its complicated role in cancer progression, and warrants further investigation.

## Impact of the overabundance of specific APC substrates on cancer behavior

All APC substrates identified in Figure 4 are individually implicated in tumor development, and many are frequently found to be overexpressed in a variety of cancers (60 of 69 proteins in Figure 4) [47, 49, 198, 199]. These discrete substrates have typically been considered in isolation, rather than as a population of APC substrates as a whole. As detailed below, the combined effect on cell biology with the accumulated overabundances of multiple APC targets includes loss of cell cycle regulation, introduction of promiscuous cycle progression, impaired apoptosis and increased genomic instability. These are classic features of cancer.

### Securin

Degradation of Securin is necessary for mitotic progression, and overexpression is a prognostic marker for worsened patient outcomes [49, 198]. Accumulation of Securin can arise from multiple mechanisms. The *hPTTG1* gene, encoding Securin, is a downstream target of estrogen receptor (ER) activation, and estrogen receptor positive (ER^+^) breast related cancers experience elevated Securin synthesis [198]. Securin accumulation may also occur as a result of selected mutations preventing Securin degradation. A specific mutation which results in this phenomenon is a T60A mutation, where threonine 60 (T60) is a crucial phosphorylation site. Substitution of the T60 amino acid prevents a destabilizing phosphorylation event, resulting in delayed, but eventual degradation of Securin [200]. Elevated Securin levels in general, but also resulting from the T60A mutation, result in increased instances of aneuploidy and chromosomal instability, identifying Securin as an important protein requiring tight regulation. Chromosomal defects are achieved by the accumulated Securin protein inhibiting proper chromosomal segregation through Separase inhibition, despite mitotic progression. Securin accumulation also results in elevated instances of cancer metastasis [49, 198].

### PLK1

Polo-like kinase 1 (PLK1) is a serine/threonine kinase that is implicated in tumorigenesis and serves as a prognostic marker for worsened patient outcomes in multiple cancers, including non-small cell lung cancer (NSCLC) [201], head and neck squamous cell carcinomas [202], and breast cancer [48, 203, 204]. Evolutionarily conserved PLK1 function is important for mitotic progression and exit; PLK1 (Cdc5 in yeast) phosphorylation targets include the APC subunits APC1, APC6, and APC3, and this is important for APC activation [205, 206]. PLK1 also phosphorylates the APC inhibitor EMI1 and inhibits the SAC (reviewed in [207]). Phosphorylation of EMI1 generates a phospho-degron motif that targets EMI1 for SCF^β-TRCP^-mediated degradation, thereby alleviating APC inhibition. It was also observed that expression of hyperactive PLK1 bypassed the mitotic block induced by nocodazole, which could be restored if a non-degradable Cyclin B1 was expressed. This suggested that hyperactive PLK1 induces a spindle checkpoint failure and prematurely activates the APC. On the other hand, normal PLK1 activity functions to promote numerous processes including chromosomal segregation, cytokinesis, mitotic entry and centrosome maturation [208–211]. A prevalent phosphorylation event performed by PLK1 is on the Cohesin protein to assist Separase in cleaving the cohesion chromatin complex [160]. Errant PLK1 activity in cancer also results in impaired apoptotic pathways [212] and *PLK1* overexpression actively promotes tumor formation after induction of DNA damage [213].

### Aurora A and B kinases

The Aurora A and B kinases have different targets, yet both phosphorylate proteins that promote chromatid segregation during cell division [214]. In multiple malignancies including colorectal [199], breast [215], pancreatic [216], and laryngeal [217] gene amplification and subsequent overexpression of Aurora A and B have been detected. Overexpression of either kinase induces chromosomal instability and tumorigenesis [215, 217], while Aurora A specifically has been found capable of overriding the mitotic arrest induced by SAC through its inhibitory phosphorylation of the BUB1 subunit, and causing mitotic slippage [218, 219]. Cancer cells are often observed to undergo mitotic slippage to avoid cell death when treated with mitotic blockers [221]. Furthermore, overexpression of Aurora A results in the aberrant phosphorylation of p73, a tumor suppressor with similarities to p53 [222, 223]. Phosphorylation of p73 by Aurora A inhibits p73 by triggering its nuclear exclusion, thereby preventing p73 from activating normal apoptotic pathways in response to DNA damage. Phosphorylation of p73 also results in further reduction of SAC activity, promoting mitotic slippage. This arises from p73-phospho-dependent dissociation of the MCC-CDC20 complex while cells are undergoing mitosis [224]. Aurora B has the opposing effect with regards to mitotic slippage, where it inhibits mitotic slippage by destabilizing kinetochores of improperly aligned chromosomes [225, 226]. The cumulative effects of the overactivity of Aurora kinases results in resistance to multiple chemotherapeutics including cisplatin and paclitaxel [218, 219, 224].

### NEK2A

NIMA related kinase 2A (NEK2A) is a splice family-member of serine/threonine kinases whose normal function is to promote the separation of centrosomes [84]. NEK2A accumulation serves as a prognostic marker for poor patient outcomes, promotes cancer cell proliferation, and is found to be upregulated across a multitude of cancers including prostate, breast, colorectal, cervical, hepatocellular carcinoma, and lung cancer [227]. NEK2A-dependent phosphorylation during mitosis serves to destabilize its targeted proteins, including centrosome linker proteins and microtubule stabilizing proteins [228, 229]. Upregulated NEK2A activity results in centrosomal defects and chromosomal instability, a hallmark molecular marker of cancer development [230, 231]. Increased NEK2A activity can also contribute to chemotherapy resistance, as NEK2A accumulation promotes ABC transporter activity through phosphorylation, as well as correlates with elevated expression of ABC transporters, themselves associated with multiple drug resistance [227].

### SNON

SNON (SKI Novel, SKIL) is targeted for degradation by the APC^CDH1^ during interphase and its overabundance contributes to tumorigenesis, owing to its ability to inhibit transforming growth factor β (TGFβ) pathways [232, 233]. TGFβ signaling pathways impact a wide variety of processes in healthy cells to prevent cell division, induce apoptosis, promote cellular differentiation, and homeostasis. However, errant TGFβ signaling, including both over and under activity, results in cancer development and progression. Overactivity of TGFβ pathways promotes the epithelial-mesenchymal transition, a key mechanism in the development of cancer [234–236]. Meanwhile, underactivity permits cancer progression [236]. Normal SNON activity acts to block TGFβ pathways prior to TGFβ activity via inhibition of SMAD2 and SMAD4, which are activated by TGFβ. After the binding of TGFβ to its targeted receptors and initiating its signaling pathways, SNON is targeted for degradation in a negative feedback loop by newly activated SMAD3 [232, 233]. During tumor progression, the overaccumulation of SNON prevents this negative-feedback from TGFβ, as SMAD3 is unable to sufficiently suppress SNON activity. The net result is that TGFβ signaling pathways remain impaired, permitting cancer progression [232–233]. SNON overexpression also specifically contributes to ER^+^ breast cancer development as SNON acts to enhance ER signaling pathways. To act in this manner SNON binds ERα-subunits that have translocated to the nucleus and enhances ERα transcriptional activity [237].

### FOXM1

The protein Forkhead Box M1 (FOXM1) is a member of the Forkhead Box (FOX) transcription factor family and primarily serves to promote the cell cycle and proliferation [238, 239]. Normal FOXM1 activity advances the cell cycle at the G1/S and G2/M transitions by transcribing genes that encode proteins that inhibit cell cycle blockers. One prominent example of this mechanism is the promotion of transcription of genes encoding the SKP2 and CKS1 proteins, subunits of the SCF E3 ubiquitin ligase [240]. Targets of the SCF include prominent tumor suppressors such as p21^Cip^ and p27^Kip^ that act to inhibit a variety of CDK proteins to prevent cell cycle progression through the G1/S transition [240, 241]. By driving the synthesis of SCF components (SKP2 and CKS1), FOXM1 initiates its own destruction, as 2 different SCF complexes, SCF^FBXL2^ and SCF^FBXO31^, target FOXM1 for degradation, with SCF^FBXO31^ specifically targeting FOXM1 at the G2/M boundary and SCF^FBXL2^ targeting FOXM1 in gastric cancer cells [242, 243]. Interestingly, FOXM1 also transcribes a number of APC substrates and activators to enter mitosis (such as CDC20, Cyclin B1, Cyclin B2, and CDC25B) [24, 244–248]. Like the SCF, activation of the APC also initiates FOXM1 destruction, as APC^CDH1^ targets FOXM1 for degradation at mitotic exit [164, 249]. FOXM1 levels are therefore heavily monitored and regulated. Elevated levels of FOXM1 are generally found in normal rapidly dividing cells [239, 240] and because of this, FOXM1 has received significant attention for its role in tumorigenesis; notably, its overexpression serves as an important prognostic marker for poor patient outcomes [250–253]. Errantly elevated FOXM1 activity has been linked to cancer metastasis [254], inhibition of apoptotic pathways [255, 256], and improper cell proliferation [257, 258]. On the hand, loss of FOXM1 resulted in prolonged G2 and delayed entry into mitosis, with an accompanying increase in aneuploid cells composed of chromosomes numbers ranging from 20-160 [246].

### CDC6

CDC6 contributes to the regulation of DNA replication as part of the DNA origin recognition complex (ORC) along with CDT1 [184, 259]. CDC6 assists in the loading of MiniChromosome Maintenance proteins 2-7 (MCMs) onto the ORC [260, 261], and is required for DNA replication [262]. CDC6 in human cells is targeted for degradation by the ACP^CDH1^ complex [263–265], whereas in yeast it appears that Cdc6 degradation requires the SCF^Cdc4^ complex [266, 267]. Even though Cdc6 degradation appears distinct between humans and yeast, its importance in DNA replication remains a commonality. MCM complexes serve as origins of replication for DNA [268], recruiting DNA stability proteins [269], and interact with the DNA repair proteins ATM and ATR to facilitate repair [270]. Impaired MCM activity results in genomic instability and an exacerbated S phase [271]. Aberrant MCM activity also results in inappropriate DNA synthesis and cellular replication [272]. Overexpression of CDC6 is often detected simultaneously with elevated CDT1 and MCM expression in a variety of cancers [273–277]. It has been established that the combined overexpression of CDC6 (both independent of, and in conjunction with, CDT1) and MCM2-7 levels correlate with poor patient prognosis in breast cancer [275]. Opposed to observations made when CDC6 is overexpressed, inappropriate CDC6 depletion subsequently results in centrosome over-duplication and premature chromosomal segregation [278].

### Geminin

Geminin plays a multifaceted role in impacting cancer development when overexpressed or overabundant. Its normal functions include binding, stabilization and inhibition of CDT1 to prevent improperly timed DNA synthesis [197, 279, 280]. Proper quantities of Geminin are necessary to protect the genome from re-replication by CDT1 [280]. Geminin is degraded by APC^CDH1^ during mitosis and G1, but during S and G2 when the APC is inactive, Geminin can begin to accumulate [281, 282]. Upon accumulation, Geminin will bind and inhibit CDT1 [279]. Due to this function, Geminin interacts with and downregulates the CDT1/CDC6 MCM pathways mentioned above. When overexpressed in cancer, Geminin promotes metastasis [274, 283], and results in poorer patient outcomes [284, 285]. It should be noted that while over-abundance of any one of these APC substrate proteins is associated with cancer development/progression, defects to APC function may lead to the over-abundance of the majority of them. This holds the potential for the development of devastating disease states.

## Contribution of APC defects to a dysregulated cell cycle

Studies supporting the necessity for the precisely timed cell cycle stages through target degradation by the APC have been carried out, indicating how APC disruptions may lead to cancer [26–32]. The three principle roles of the APC regarding control of the cell cycle include promoting mitotic progression (or inducing mitotic slippage), regulating the entrance to S phase, and inducing cell cycle arrest [16, 67, 183, 185, 193, 286, 287].

### Mitotic slippage

Incongruous and/or sustained SAC activation causes mitotic arrest [63, 214]. However, after prolonged arrest some cells can undergo an uncontrolled mitotic progression referred to as mitotic slippage, generating a potential chemotherapy-resistant state in those cells able to pass through this checkpoint inappropriately [220, 221, 288, 289]. There are multiple common consequences to mitotic slippage. First, the cell is likely to proliferate in an unregulated manner [290]. Mitotic slippage can also result in increased chromosomal damage and mis-segregation [291, 292]. Lastly, mitotic slippage induces resistance to chemotherapies disrupting microtubule formation (chemotherapeutics such as Paclitaxel falls under this category). This is due to microtubule poisons relying on prolonging SAC activity in cells that do not carry a heavy load of chromosome instability, but to the point of triggering mitotic slippage, a mechanism dependent on APC driving mitosis despite SAC activity [219, 291, 293]. On the other hand, in cells harboring high loads of chromosomal instability due to excess DNA mutations, induction of mitotic slippage has been proposed as a mechanism to kill these cells. Chemicals that inhibit the SAC, such as TTKi’s [294–296] and M2I-1 [58, 62], have been shown to block CDC20 sequestration by the MCC, leading to activation of the APC and effective cancer cell death. It is proposed that premature activation of the APC pushes cells with high loads of chromosome instability into mitotic division before there is time to repair the damage, causing mitotic catastrophe [31].

Improper regulation of the APC can induce mitotic slippage through multiple mechanisms. First, the overexpression of *CDC20*, as described above, prevents the SAC from inhibiting APC activity due to an inability to sufficiently sequester the excess CDC20 protein. This allows the unsequestered APC^Cdc20^ to promote anaphase, with mitotic slippage occurring as a result [174, 175]. However, it should be noted that while this is a possibility, enhanced APC activity and anaphase progression should, in the end, result in elevated targeting of CDC20 for degradation. A second mechanism of the APC overcoming SAC inhibition is through CDH1 activity. As the SAC cannot directly inhibit CDH1 activity, failure of the regulatory mechanisms that inhibit APC activation via CDH1 results in mitotic slippage, as APC^CDH1^ can prematurely target Securin for degradation [193, 194]. This occurs principally if Cyclin B1 activity is impeded, as CDK1^Cyclin B1^ phosphorylation of CDH1 prohibits binding to the APC. This dysfunction may occur if there is insufficient Cyclin B1 expressed during mitosis, or if there is a deficiency of ATP which is necessary for CDK1 to perform its phosphorylation events [286]. Aurora A, when in abundance, is also capable of inducing mitotic slippage through inhibition of SAC [218, 219].

### Regulating S phase entrance

APC^CDH1^ plays a crucial role in regulating the entrance to S phase. During mitosis a failure to degrade the mitotic Cyclins A and B results in the proteins improperly accumulating in G1 and results in a premature promotion of S phase [75, 186]. APC^CDH1^ also directly regulates entry to S phase, in conjunction with the SCF. Depletion of CDH1 results in premature entry to S phase, as well as a prolonged S phase [66, 75, 182]. APC^CDH1^ and SCF^Cyclin F^ form a double negative feedback loop, where APC^CDH1^ targets Cyclin F for degradation, and SCF^Cyclin^ ^F^ targets CDH1 for degradation [67]. Coupled with the negative feedback loop of APC^CDH1^ autoubiquitination of CDH1 [189], expression of Cyclin F and formation of SCF^Cyclin F^ during G1 reaches a critical point of CDH1 depletion where APC^CDH1^ activity is unable to prevent full SCF^Cyclin F^ activity and the subsequent transition to S phase. Knockout of Cyclin F using siRNA resulted in a prolonged G1, however simultaneous siRNA knockout of CDH1 reversed this phenotype [67]. The timed degradation of CDH1 created by this mechanism permits a regulated entry to S phase, as loss of APC^CDH1^ activity results in the accumulation of Cyclin A [67]. APC^CDH1^ also polyubiquitinates the SCF subunit SKP2 for degradation to prevent cell cycle progression [32, 183]. APC^CDH1^ can also delay entry to S phase via polyubiquitination and subsequent degradation of the proliferating cell nuclear antigen (PCNA) associated PAF15 [111].

### Inducing cell cycle arrest

The APC^CDH1^ complex can initiate cell cycle arrest at multiple stages of the cell cycle [186]. At the G2/M transition, APC^CDH1^ acts in conjunction with CDC14B and PLK1 to prevent progression into mitosis in the event of DNA damage [177]. In response to DNA damage that occurs during the G2/M transition, the phosphatase CDC14B translocates to the nucleoplasm from the nucleolus and activates APC^CDH1^ via removal of inhibitory phosphorylation events blocking recruitment of CDH1 to the APC. APC^CDH1^ will then target PLK1 for degradation, resulting in transient stabilization of Claspin, a protein required for the initiation of DNA repair pathways [177]. Once the checkpoint is satisfied, phosphorylation of Claspin by residual PLK targets it for SCFβ^-TrCP^-mediated degradation [297–299]. Under normal conditions, it has been shown that Claspin is targeted by ACP^CDH1^ during G1 [177].

Genotoxic stress is not the only stressor that activates cell cycle arrest through the APC. Nutrient stresses also activate cell cycle arrest through the APC [16, 186, 260]. In CDH1^-/-^ chicken cells (DT40) rapamycin is unable to induce G1 cell cycle arrest [186]. This is a result of altered CDK2 and retinoblastoma (Rb) pathways. Upon rapamycin treatment, wild type cells lose Rb phosphorylation, allowing the induction of G1 arrest, but in CDH1^-/-^ cells, Rb phosphorylation is maintained with continued cell cycle progression [186]. In *S. cerevisiae*, Cdh1 acts to protect the cell from ethanol, caffeine, and hyperosmotic stress, as yeast cells lacking *CDH1* still progress through the cell cycle, but are sensitive to multiple stresses [16]. The stress sensitivity appears to be due to elevated stability of Clb2 (orthologous to human Cyclin B2) and Hsl1 (ortholog of human NIM1-related Kinase) from a partially impaired APC that continues to drive cells through the G2/M transition despite the incurred cellular damage. Meanwhile, inhibition of the APC in quiescent cells drives their return to the cell cycle [26, 260]. This indicates that APC activity is required both for entrance to, and maintenance of, cell cycle arrest.

Acetylation of both CDC20 and CDH1 are key regulatory events impacting APC activity, as it prevents their respective bindings to the APC [65]. A lack of deacetylation of these APC coactivators, due to loss of the SIRT2 deacetylase, leads to elevated APC inhibition and lack of target degradation. This ultimately results in enhanced abundance of APC substrates, abnormal amplification of centrosomes, increased aneuploidy events and eventually mitotic catastrophe [65]. Studies in *S. cerevisiae* have revealed the complicated networks that the deacetylation enzyme Sir2, the yeast orthologue of SIRT2, impacts. Sir2 is an important stress response and longevity protein in *S. cerevisiae*, and it is tightly connected with a stress response network that interacts with the APC, namely the Fkh1 and Fkh2 Fox transcription factors [103, 104, 300]. In *S. cerevisiae*, under stress conditions, the APC and Fkhs work together to induce a response to stress [18, 20, 24]. Furthermore, when stress is encountered, Sir2 is recruited to Clb2 promoters in a Fkh1-dependent manner to repress *CLB2* expression and stall the cell cycle [300]. Therefore, SIRT2 may be part of the mammalian APC stress response network, and thus a key regulator of the cell cycle.

## APC subunit mutation

The notion that the APC is primarily important for cell health and avoidance of cancer progression suggests that loss of APC subunits may be linked to cancer development or progression. However, complete loss of APC function in animals is lethal [287, 301]. With this in mind it is not surprising that APC subunit mutations are rarely reported in animal and cell systems [302]. Nonetheless, APC subunit mutations have been reported, as briefly discussed above. For example, APC5 and APC7 were shown to interact with the CBP/p300 transcriptional activator, a histone acetyltransferase, and to play a direct role in transcriptional activation [303]. CBP/p300 is targeted by E1A to induce tumorigenic transformation. Further analysis showed that overexpression of APC5 or APC7 suppressed the transformative ability of E1A, while knockdown of APC5 or APC7 *in vitro* resulted in enhanced transformation, highlighting the role of the APC in stalling tumor transformation. Other studies have shown that APC7, and APC16 (subunits not observed in yeast) form a complex with APC3 [304]. Deletion of APC7 or APC16 in HCT116 colon cancer cells, however, revealed no overt phenotypes other than reduced *in vitro* ubiquitination activity [57]. These studies showed, nonetheless, that in APC7 or APC16 deletion cells, ablation of the essential MAD2 was tolerated. These cells had accelerated mitosis, no longer responded to SAC activity, and sustained increased genomic instability. The importance of APC7 was further suggested when 108 invasive ductal breast carcinomas were stained for APC7 expression [305]. It was reported that loss of APC7 was predominantly found in cases with poor prognosis or signs of malignancy. In other studies, it was found that Rothman-Thomson Syndrome Type 1, which causes juvenile cataracts, is due to a premature stop codon in APC1, resulting in reduced, but not complete loss of APC1 protein [306]. Additional studies revealed mutations in CDC16 and CDC23 in human colon cancer cells [56]. Interestingly, opposed to studies showing loss of APC subunit functions in many cancers, increased APC11 mRNA was observed in colorectal cancer samples, and correlated with worse overall survival [307]. APC11 is the APC catalytic subunit, so it remains a question as to why this subunit would behave differently than the other subunits studied in regards to cancer. Taken together, the bulk of the evidence indicates that mutations to variety of APC subunits confers a risk for disease onset.

## CONCLUSIONS

Through its interactions with numerous cellular pathways, the APC maintains a complicated position in cancer development. While bound to CDC20, it acts in an oncogenic fashion and promotes tumor development; however, when bound to CDH1, the APC displays many tumor suppressive effects (Figure 4). Many genes encoding protein substrates normally degraded by APC E3 activity are found to be overabundant in a wide variety of cancers. Furthermore, many of the phenotypes associated with defective APC activity, such as elevated genomic instability, improperly regulated cell cycle, and aneuploidy, contribute to tumor progression and drug resistance. This suggests that activation of the APC, as previously suggested for prolonging lifespan [23], may also be relevant for treating cancer. Targeting APC activity has shown promise in an anti-tumor capacity, as the SAC inhibitors M2I-1 and TTKi, which both disrupt CDC20-SAC interactions, increase APC^CDC20^ activity and provide increased killing of cancer cells [31, 58–62]. In our current work we have observed loss of APC activity in canines with drug resistant lymphoma, and that increased APC activity was associated with remission, and APC activity loss again occurred when the animal relapsed (Arnason et al. under review). Furthermore, loss of SIRT2 and the resulting impaired activity in both APC^CDH1^ and APC^CDC20^ complexes, or the loss of CDH1 itself, promotes genomic instability and tumor progression [65], indicating that generalized APC dysfunction is tumorigenic [26–31]. Moreover, numerous reports have now identified a signature of overexpressed genes that encode APC substrates and inhibitors in a variety of aggressive tumors [37, 47]. Taken together, this provides a compelling rationale to further research directed at the role the APC plays in tumoral development.

## Supplementary Material

Supplementary Figures
