# Supplementary material for: The role of Anaphase Promoting Complex activation, inhibition and substrates in cancer development and progression
Source: Aging (Albany NY). 2020 Aug 15;12(15):15818–55. doi: 10.18632/aging.103792 (PMC7467358; doi:10.18632/aging.103792)
Supplement: Supplementary Figures [file aging-12-103792-s001..pdf]

SUPPLEMENTARY FIGURES

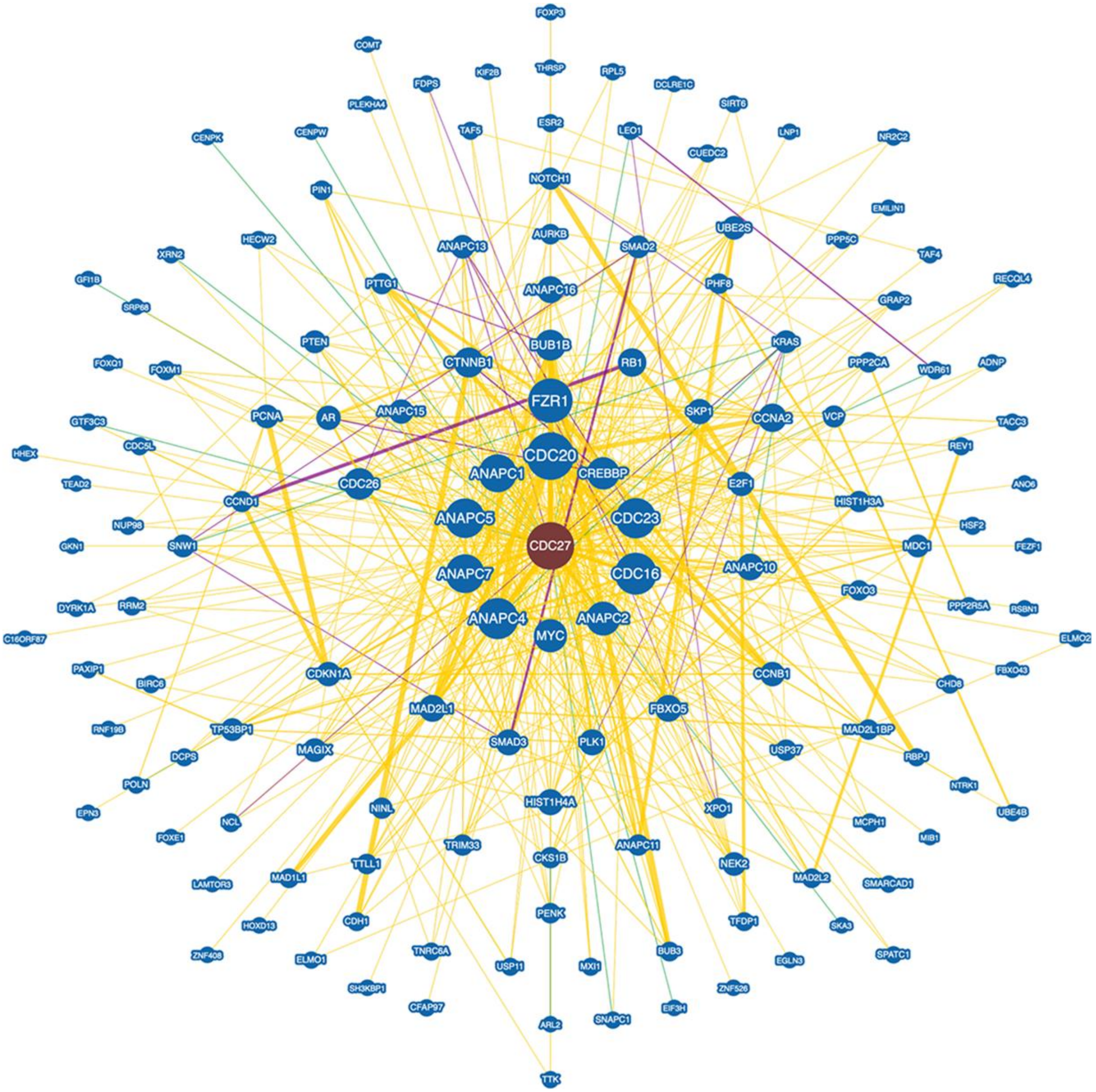

Supplementary Figure 1. Raw data from a BioGRID search using the search word CDC27. Minimal evidence was set to 1 for all searches. Nonhuman interactors and homologous were removed from the results. The same criteria was used for all searches.

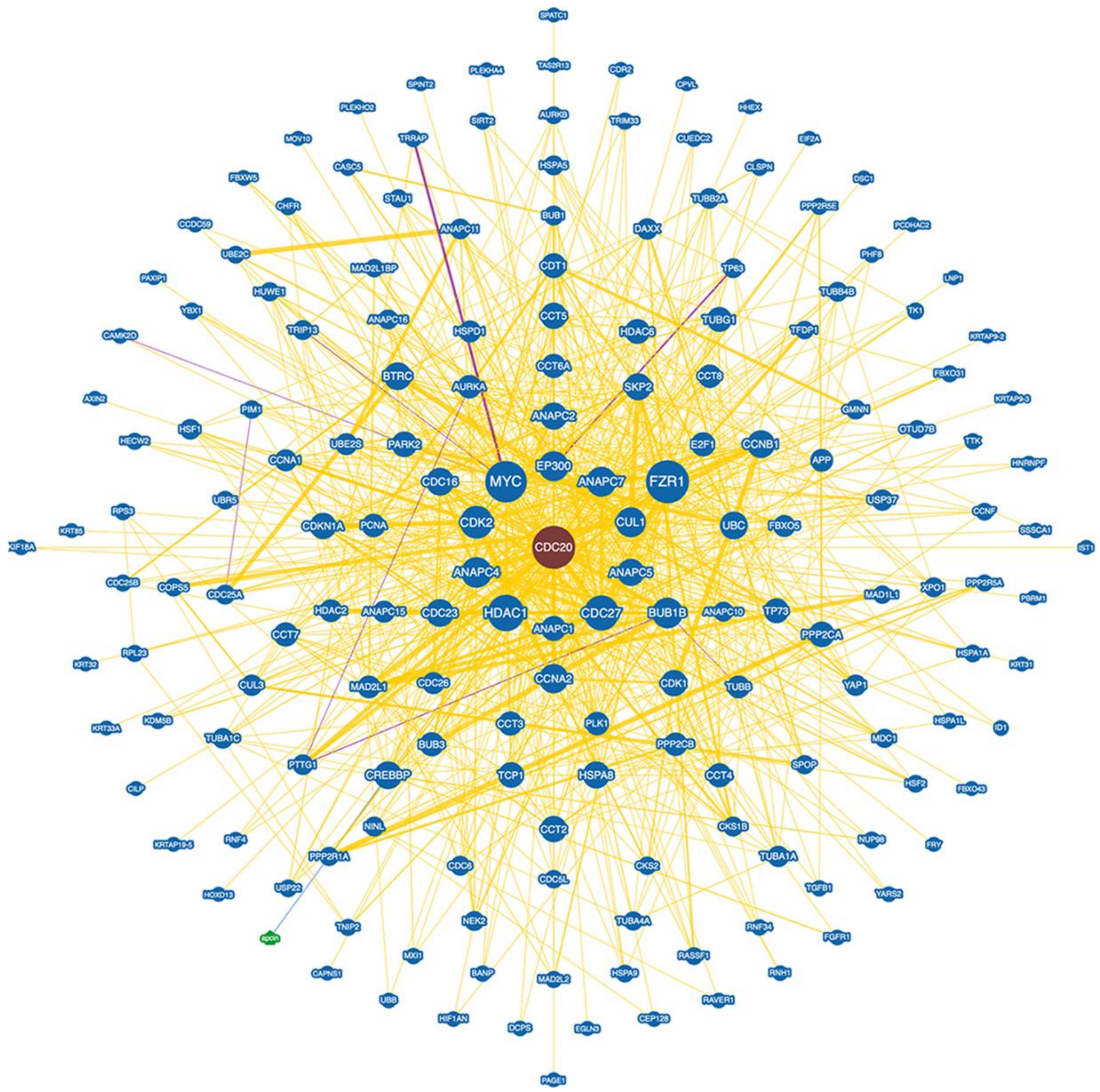

Supplementary Figure 2. Raw data from a BioGRID search using the search word FZR1.

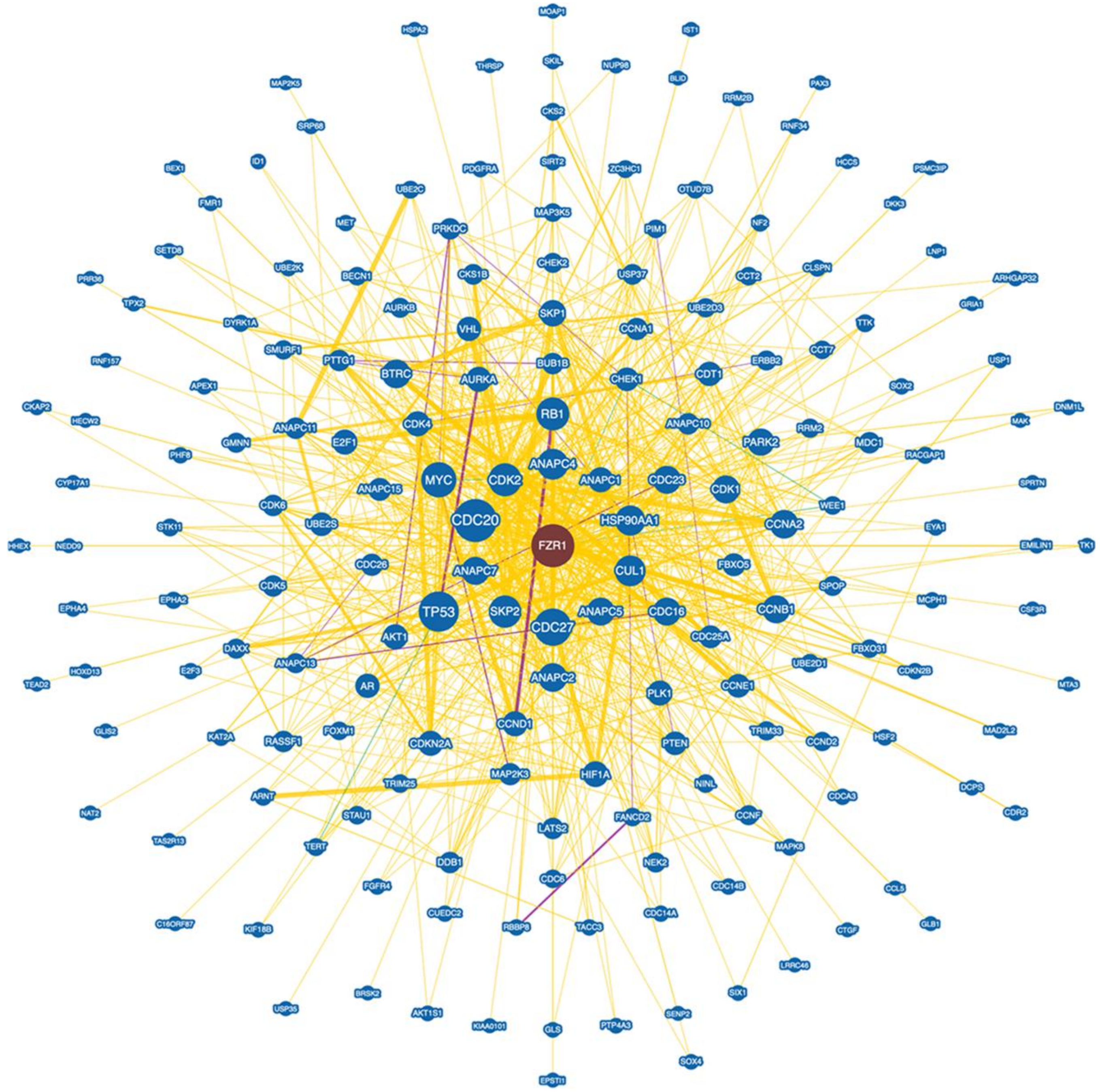

Supplementary Figure 3. Raw data from a BioGRID search using the search word CDC20.
